# Supplementary material for: Improvement of Selected Quality and Safety Traits in Turmeric-Enriched Kale Pesto Using Blue Light and Sous-Vide
Source: Molecules. 2024 Dec 11;29(24):5831. doi: 10.3390/molecules29245831 (PMC11728637; doi:10.3390/molecules29245831)
Supplement: Supplementary file 1 [file molecules-29-05831-s001.zip › molecules-3310490-supplementary.pdf]

Article

Improvement of Selected Quality and Safety Traits in Turmeric-enriched Kale Pesto using Blue Light and Sous-Vide

Magdalena A. Olszewska [1,*], Anna Draszanowska [2*], Aleksandra Zimińska [1], and Małgorzata Starowicz[3]

[1] Department of Food Microbiology, Meat Technology and Chemistry, The Faculty of Food Science, University of Warmia and Mazury in Olsztyn, Plac Cieszyński 1, 10-726 Olsztyn, Poland; aleksandra.ziminska@student.uwm.edu.pl
[2] Department of Human Nutrition, The Faculty of Food Science, University of Warmia and Mazury in Olsztyn, Słoneczna 45F, 10-718 Olsztyn, Poland
[3] Department of Chemistry and Biodynamics of Food, Institute of Animal Reproduction and Food Research of Polish Academy of Sciences, Juliana Tuwima 10, 10-748 Olsztyn, Poland; m.starowicz@pan.olsztyn.pl
* Correspondence: magdalena.olszewska@uwm.edu.pl (M.A.O.); anna.draszanowska@uwm.edu.pl (A.D.)

Table S1. The CIELAB color space conversion to RGB color model in which red, green and blue are added to reproduce colors.

**Processing step**	**Day 0**		**Day 14**	
	**w/o Turmeric**	**w/ Turmeric**	**w/o Turmeric**	**w/ Turmeric**
Raw	73, 95, 47*	71, 92, 37	67, 87, 22	73, 94, 21
Sous Vide	75, 96, 45	75, 96, 35	78, 91, 35	86, 100, 41
Blue Light	68, 81, 44	69, 84, 44	71, 81, 45	69, 80, 36
Blue Light and Sous Vide	73, 86, 50	76, 89, 48	72, 79, 43	77, 83, 42

* red, green, blue coordinates

Materials and Methods

1. *Moisture Content*

Moisture content in kale pesto samples was determined by drying to constant weight at 105 °C according to AOAC procedure No. 934.01 (AOAC, 2005) using a forced draught laboratory oven (UF55; Memmert, Schwabach, Germany).

AOAC. Official methods of analysis method (2005) 934.01, 18th Ed., AOAC INTERNATIONAL, Gaithersburg, MD.

Table S2. Results of the moisture content of minimally processed kale pesto with and without the addition of turmeric.

	**Pesto version**
**Processing step**	**w/o Turmeric**
Raw	81.32±0.96
Sous Vide	80.15±0.54
Blue Light	74.65±0.57
Blue Light and Sous Vide	74.66±0.07
**Processing step**	**w/ Turmeric**
Raw	79.54±0.03
Sous Vide	80.29±0.07
Blue Light	75.06±0.35
Blue Light and Sous Vide	74.15±0.24
